# Modifications of Visual Field Asymmetries for Face Categorization in Early Deaf Adults: A Study With Chimeric Faces

**DOI:** 10.3389/fpsyg.2017.00030

**Published:** 2017-01-20

**Authors:** Marjorie Dole, David Méary, Olivier Pascalis

**Affiliations:** ^1^Laboratoire de Psychologie et NeuroCognition, CNRS UMR 5105, Université Grenoble-AlpesGrenoble, France; ^2^Gipsa-Lab, Département Parole et Cognition, CNRS UMR 5216, Université Grenoble-AlpesGrenoble, France

**Keywords:** early deafness, hemispheric laterality, chimeric face, gender, eye movements, categorization task

## Abstract

Right hemisphere lateralization for face processing is well documented in typical populations. At the behavioral level, this right hemisphere bias is often related to a left visual field (LVF) bias. A conventional mean to study this phenomenon consists of using chimeric faces that are composed of the left and right parts of two faces. In this paradigm, participants generally use the left part of the chimeric face, mostly processed through the right optic tract, to determine its identity, gender or age. To assess the impact of early auditory deprivation on face processing abilities, we tested the LVF bias in a group of early deaf participants and hearing controls. In two experiments, deaf and hearing participants performed a gender categorization task with chimeric and normal average faces. Over the two experiments the results confirmed the presence of a LVF bias in participants, which was less frequent in deaf participants. This result suggested modifications of hemispheric lateralization for face processing in deaf participants. In Experiment 2 we also recorded eye movements to examine whether the LVF bias could be related to face scanning behavior. In this second study, participants performed a similar task while we recorded eye movements using an eye tracking system. Using areas of interest analysis we observed that the proportion of fixations on the mouth relatively to the other areas was increased in deaf participants in comparison with the hearing group. This was associated with a decrease of the proportion of fixations on the eyes. In addition these measures were correlated to the LVF bias suggesting a relationship between the LVF bias and the patterns of facial exploration. Taken together, these results suggest that early auditory deprivation results in plasticity phenomenon affecting the perception of static faces through modifications of hemispheric lateralization and of gaze behavior.

## Introduction

Hemispheric specialization of cognitive function in typical adult brain is well documented. One well-known example is the lateralization of language in the left hemisphere, perisylvian areas dedicated to language processing being functionally (see Tzourio et al., 1998; Celsis et al., 1999, for examples) as well as anatomically (Geschwind and Levitsky, 1968; Foundas et al., 1995) left lateralized in the great majority of right-handed subjects. Conversely visuo-spatial and face processing abilities would be lateralized toward the right hemisphere. This right hemisphere dominance for face processing has been originally demonstrated, thanks to prosopagnosic patients – patients showing specific inability to recognize faces following brain damage. Indeed, although prosopagnosics generally suffer from bilateral lesions, a right hemisphere lesion seems sufficient to produce significant impairments in face recognition (De Renzi, 1986; De Renzi et al., 1991). The hypothesis of a right hemisphere advantage for face processing is also supported by fMRI results, showing a functional asymmetry in favor of the right hemisphere during face processing, particularly in the Fusiform Face Area (FFA; Badzakova-Trajkov et al., 2010; Rossion et al., 2012; Bukowski et al., 2013).

At the behavioral level, this right hemisphere dominance for face processing is thought to be the cause of a left visual field (LVF) bias, the fact that facial information present in the LVF is crucial for categorization and recognition (Levy et al., 1983; Luh et al., 1991; Burt and Perrett, 1997). Burt and Perrett (1997), for example, used chimeric faces (faces vertically split in two different halves) to assess right hemisphere advantage during the detection of variable face attributes, such as gender, age or facial expression. The stimuli presented to participants were composed of two average half faces (e.g., left half is an average of male faces whereas right half is an average of female faces) with the join down the center blended rendering it invisible to participants. The rationale beyond this image manipulation is that the left and right hemispheres receive respectively the right and left part of the image relative to the point of foveation. Gazing three degree to the right of a face will place the entire image in the LVF and this signal, conveyed through the right visual tract, will be first processed in the right hemisphere. Acuity drops drastically with eccentricity from point of foveation and we generally look directly at faces to access to more details. Nevertheless, when we fixate different locations in a face, the left and right hemisphere are processing only partly overlapping right and left parts of the face. Burt and Perrett (1997) found that participants’ judgments of gender and expression were influenced to a greater extent by the information on the left of the face from the viewer’s perspective. This finding has been largely replicated (Butler and Harvey, 2005; Butler and Harvey, 2008; Yovel et al., 2008; Bourne and Gray, 2011). To establish a straight relationship between hemispheric lateralization and the LVF bias obtained using chimeric faces, Yovel et al. (2008) used fMRI while participants performed a matching task. Participants were presented chimeric faces in the scanner, and also performed the same task outside the scanner. The resulting activity in the FFA was rightward asymmetric, and this asymmetry was positively correlated with the LVF bias obtained from the behavioral test ran outside the scanner. This confirmed that the LVF bias obtained using chimeric faces does, at least in part reflect right hemispheric specialization of face processing areas. Right hemisphere advantage for the processing of face could be related to the processing of configural information in face (Schiltz and Rossion, 2006; Maurer et al., 2007), right hemisphere being generally thought to process predominantly global information whereas left hemisphere would be specialized in the processing of local information (Fink et al., 1996, 1999; Lux et al., 2004).

The relative contribution of right hemisphere lateralization and attentional factors resulting from the scanning patterns in the LVF bias is, however, not fully understood. An increased LVF bias in trials in which participants spent more time looking at the left part of the face suggests a clear link between attentional factors resulting from scanning patterns and the LVF bias (Butler et al., 2005). However, this bias can be observed even with short presentation times (100 ms) that are preventing eye movements, ruling out the effect of purely attentional factors (Butler and Harvey, 2006, 2008). The presence or absence of a LVF bias would thus result from a complex interplay between bottom-up perceptual processing factors and top–down attentional factors which could be both lateralized, similarly to what has been suggested for written language (Selpien et al., 2015).

The LVF bias is also robustly found even with line drawing (Luh et al., 1991), or inverted stimuli (Butler and Harvey, 2005). Using chimeric faces, Aljuhanay et al. (2010) found that the LVF bias was present as early as 5 years of age. The root of the hemispheric asymmetry in face processing has been hypothesized to lie in the development of the hemispheric specialization. Infants recognize a face faster if it is initially presented in the LVF as opposed to the right visual field (de Schonen and Mathivet, 1990). This processing bias may represent the precursor of the asymmetry observed in adults.

If the brain asymmetry for face processing emerges during development, early deprivation or dramatic differences the infant experiences with the world should affect it. For example, 9- to 23-year-old participants treated for bilateral congenital cataracts after 7 weeks of age, who were deprived of patterned visual input during this duration, fail to develop some of the aspects associated with typical adult levels of face recognition such as the face composite effect suggesting impaired configural processing (Le Grand et al., 2001). This emphasizes the importance of early visual experience in the development of adult face processing abilities. Cross-modal interactions have also been found to affect visual development; for example early auditory deprivation has been shown to affect the development of some visual abilities. Several studies showed that deaf participants could detect targets at larger eccentricities, indicating larger visual field (Buckley et al., 2010; Codina et al., 2011). Better abilities have also been found in deaf participants for the detection of motion in the visual periphery (Armstrong et al., 2002; Bosworth and Dobkins, 2002; Stevens and Neville, 2006; Hauthal et al., 2013). The observation of enhanced processing in the periphery, particularly under attentional conditions, seems very reliable in the literature (Parasnis and Samar, 1985; Neville and Lawson, 1987; Bavelier et al., 2000, 2001; Bottari et al., 2010). Higher-level visual abilities have also been shown to be modified by early deafness, such as visual imagery (Emmorey et al., 1993, 1998) or the processing of faces (Bettger et al., 1997; Arnold and Murray, 1998). Using the Benton Test of Facial Recognition, Bettger et al. (1997) tested the recognition of individual faces in deaf participants. They obtained better scores than hearing non signers, but only in a difficult condition, in which faces were shadowed. This enhanced processing in deaf people could thus concern very particular aspects of face processing; McCullough and Emmorey (1997) found that deaf and hearing participants differed only by the detection of subtle facial features. Feature analysis relates to configural face processing and de Heering et al. (2012) suggested an increased dependency on this mode of processing in deaf participants.

If visual processing is affected by early deafness, what about visual asymmetries? Several experimental studies examining hemispheric asymmetry in congenitally deaf individuals found that it differs from the one observed in hearing individuals (Szelag and Wasilewski, 1992; Szelag, 1996; Neville et al., 1997). As for the processing of sign language, an extensive amount of data show that it could activate the typical left-lateralized speech processing network (Pettito et al., 2000; MacSweeney et al., 2002, 2008). However, other studies suggest greater contribution of the right hemisphere for the processing of sign language than for spoken language (Neville et al., 1997, 1998; Emmorey et al., 2002). In addition damage to both left and right hemisphere lead to language deficit in sign language users (Corina and McBurney, 2001). Neville and colleagues proposed that greater recruitment of right hemisphere would be related to the visual-spatial characteristics of sign language. However, recent results suggest a reduction of hemispheric lateralization in a spatial attention task (Cattaneo et al., 2014). A shift of hemispheric lateralization during the detection of motion has also been demonstrated, deaf subjects showing a left hemisphere advantage, whereas hearing subjects showed a right hemisphere advantage (Bosworth and Dobkins, 1999; Bavelier et al., 2001; Bosworth et al., 2013). The question of cerebral lateralization for sign language processing results from a complex interplay between language-related and other cognitive – visuo-spatial, gestural, motion-related – functions modulated by sensory experience that are still poorly understood.

With regard to face processing, few studies investigated hemispheric lateralization during the perception of faces in deaf people and the results are rather contrasted. Phippard (1977) presented briefly unfamiliar faces in either the left or right visual field and found no differences between deaf and hearing participants. More recently Letourneau and Mitchell (2013) found a typical LVF asymmetry during an identity judgment task. Neurophysiological studies suggest a reduced right hemisphere asymmetry in deaf participants compared with hearing ones (Weisberg et al., 2012; Mitchell et al., 2013). Mitchell et al. (2013) for example found a reduced asymmetry of the neural responses to neutral faces around 200 ms in deaf participants when compared with the hearing group. Other studies principally focused on the processing of emotional and/or linguistic facial expressions. Indeed, although facial expressions are a universal cue to recognize the emotional state of individuals, sign language users must also recognize facial expressions as linguistic markers which could affect the hemispheric lateralization. Concerning emotional expressions, Szelag and Wasilewski (1992) presented emotional (happy, sad) and non-emotional faces in the left or right visual field in deaf children. They found a LVF (right hemisphere) advantage for neutral and sad faces in normal hearing, and no hemispheric advantage for any kind of faces in deaf participants. More recently, Letourneau and Mitchell (2013) found a reduction of the LVF bias during an emotion judgment task in deaf participants. Corina (1989) investigated the LVF advantage for affective and linguistic facial expressions; they obtained a reduced LVF bias in deaf participants for both types of expressions, but this was strongly affected by the order of presentation. A following study (Corina et al., 1999) suggests that the cerebral lateralization for facial expression could depend on the functional role (linguistic/affective) of these expressions. Finally, McCullough et al. (2005), using fMRI, investigated cerebral asymmetries during the presentation of linguistic or emotional facial expressions. For emotional expressions, they found a right hemisphere lateralization in the STS in hearing controls, whereas activation was symmetrical in deaf participants. For linguistic facial expressions, activation was also right lateralized in hearing subjects, but left lateralized in deaf participants. Some modifications of the asymmetry for emotional and linguistic facial expressions were also observed in the fusiform gyrus, where hearings exhibited a slight rightward asymmetry for both types of expressions, whereas activity was leftward lateralized in deaf participants (see also Emmorey and McCullough, 2009).

Taken together, these results suggest differences of the functional hemispheric asymmetry for the processing of both linguistic and emotional expressions. However, it still unclear whether this plasticity extends to the core aspects of face processing. By presenting neutral faces, Weisberg et al. (2012) found a reduced activity in the right fusiform gyrus in deaf participants in comparison with hearing non signers, whereas no difference was observed in left fusiform. This could suggest reduced asymmetry in deaf participants. However, this study being not designed to investigate cerebral asymmetry, it remains difficult to draw firm conclusion about hemispheric lateralization in deaf participants.

Our review of the literature suggests the existence of some modifications in the cerebral asymmetries in deaf people, resulting either from auditory deprivation or/and their extensive use of sign language. Concerning facial processing, these modifications are less well established. To date, evidences for modifications of hemispheric lateralization for the processing of neutral faces are rather scarce and it is unclear how deafness affects the processing of invariant aspects of face. The present experiments are interested in examining the LVF bias in a population of deaf adults and non-signer hearings. We used a gender recognition task with chimeric faces. Assuming that LVF bias reflects right hemispheric dominance for face processing we predicted a reduced LVF bias in deaf participants. From the results of Weisberg et al. (2012), this reduced LVF bias would be linked to a reduced activity in the right fusiform gyrus during face processing. This hypothesis was tested in Experiments 1 and 2. In Experiment 2 we also measured eye movements during face scanning to investigate the consequences of early auditory deprivation on the visual attention toward face features in the gender recognition task.

## Experiment 1

### Participants

Fourteen deaf adult participants (six females, mean age: 34.92, *SD*: 8.58) and 14 normal hearing controls (seven females, mean age: 31.27, *SD*: 8.56) selected to match deaf participants in gender, age, and handedness participated in the study. A two sample *t*-test confirmed that the two groups did not differ in age or handedness (both *p* > 0.05). All participants were right-handed according to the Edinburgh Handedness Inventory (Oldfield, 1971). Deaf participants were bilateral severe to profoundly deaf (80 dB hearing loss and greater) and all were prelingually deaf. None of the hearing participants were exposed to signed language. No participants reported any neurological or psychiatric illness, and all had a normal to corrected vision. Details concerning the characteristics of the deaf group can be found in **Table 1**. All participants signed written informed consent and were paid for their participation.

**Table 1:** Characteristics of the deaf group.

Gender	Male	8
	Female	6

Mean age		34.92

Origin of deafness	Congenital deafness	6
	Pregnancy related	1
	Childhood illness	1
	Unknown	6

Sign language	Yes	13
	No	1

Age of learning to sign	Before 3	3
	Between 3 and 11	7
	Between 11 and 18	2
	Adulthood	2

Lip reading	Yes	12
	No	2

Hearing aid	None	3
	One Ear	2
	Both Ears	9

Family history of deafness	Yes	4
	No	10

### Material and Procedure

**FIGURE 1:**
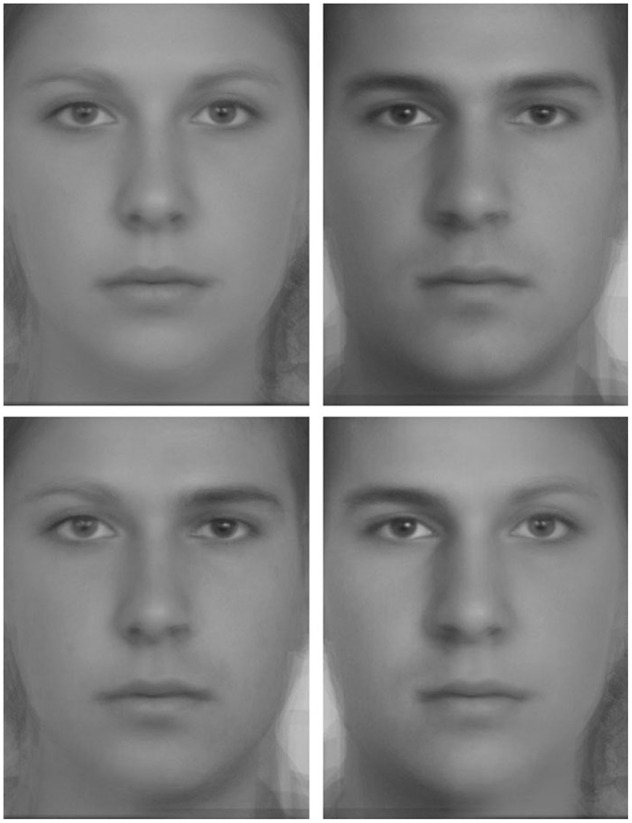
**Example of the stimuli used in this experiment. (Top)** Blended male and female faces. **(Bottom)** Chimeric female/male **(Bottom left)** and male/female **(Bottom right)** faces.

Forty faces were presented in a randomized order to the participants: 10 female/male (Chimeric F/M), 10 male/female (Chimeric M/F), 10 blended female (Entire F) and 10 blended male (Entire M) (**Figure 1**). Stimuli used were previously described (see Burt and Perrett, 1997; Butler et al., 2005). Briefly, each chimeric face was composed of one blended male and one blended female face. Each blended face was composed of five different faces with the age of photographed people approximately matched. Additional features that could facilitate gender recognition such as earrings, make-up or beard, were absent. Before blending, all faces were rotated and aligned with respect to eyes and mouth. After blending, 10 blended female and 10 blended male images were selected to create 10 pairs of chimeric faces. The two blended faces composing a pair were aligned to match eye position across the pair. The first picture of the pair was composed with the left half of the blended male face and the right half of the blended female face, and the second picture of the pair was the mirror of the first image. Gradual change in shape and color from one image to the other across the vertical midline produced a seamless merger between the left and right halves of the chimeric faces rendering the vertical midline between the two halves imperceptible. Each face was then converted from color to gray-level.

The 40 stimuli were presented centrally on an Iiyama Vision Master Pro 513 screen. Screen resolution was 1024 × 768 pixel (40.5 cm × 30 cm). Participants sat 62 cm away from the screen, their chin relying on a chin rest. Stimulus size was 396 × 522 pixels. Participants were required to indicate by key press on the keyboard if the face was feminine or masculine. All responses were made using the right hand only and the positioning of male and female labels was counterbalanced between participants. All participants performed five practice trials in order to ensure good comprehension of the instructions. Participants were given enough time to provide their answer, but were encouraged to answer as quickly as possible. The image was displayed until the participants gave their answers. Instructions were given in writing and orally for the hearing participants and in writing and either orally or in French Sign Language depending on preference for the deaf participants.

### Results

Statistical analyses were run in R (R Development Core Team, 2008). Rapid analysis of the percentage of correct gender classification for the average female and male faces showed that it was nearly perfect in both groups (deaf: 99.6%; hearing: 98.6%). The mean response time of hearing participants seemed faster than that of the deaf participants (deaf: *M* = 1070 ms, *SD* = 258 ms; hearing: *M* = 920 ms, *SD* = 241 ms) but this difference was not significant (two-sample *t*-test, *t*_26_ = -1.58, *p* = 0.12).

Response time increased in both groups when judging chimeric faces (deaf: *M* = 1768 ms, *SD* = 330 ms, paired *t*-test, *t*_13_ = -3.37, *p* = 0.005; hearing: *M* = 1332 ms, *SD* = 555 ms, *t*_13_ = -6.32, *p* < 0.001). This increase in response time reflects the increased difficulty in judging the gender of chimeric faces. We also found a significant interaction between Group and the Type of Face (Entire, Chimeric) [*F*(1,42) = 7.70, *p* = 0.01], indicating that the increased response times for the Deaf group was particularly important for chimeric faces and not for entire faces.

**FIGURE 2:**
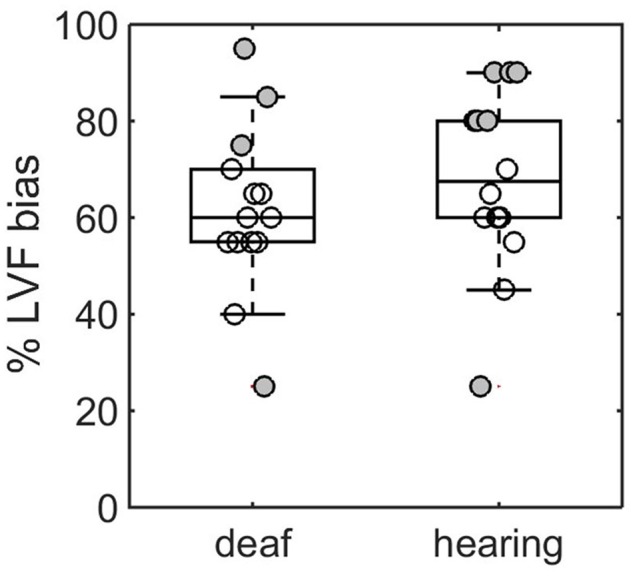
**Visual field bias for deaf and hearing participants.** Box center and limit give the median, 1st and 3rd quartile. Each data point represents a participant. Filled data points indicate significant bias according to a χ^2^ statistics. Two participants showed a RVF bias (filled dots < 50%).

For the analysis of responses, a score of 1 was given if the participant’s answer represented a LVF bias (i.e., female for chimeric F/M stimuli and male for chimeric M/F stimuli) and 0 otherwise. The average score over the 20 chimeric faces × 100 was used as an index of LVF bias with value above 50% representing a LVF bias while value below 50% represents a RVF bias. A boxplot showing the median, 1st and 3rd quartiles, and individual data points for both groups is given in **Figure 2**. First, we built a generalized linear model of LVF index. Formally the model was written LVF-50 = *β*_1_ + *β*_2_G*_j_* + *𝜀_ij_* where G represented the group and was coded *j* = 0 for hearing and *j* = 1 for deaf. We subtracted 50 from the LVF values to center the result with respect to chance level (50%). According to the model the intercept term represents the amount of LVF bias in the hearing group and the second term *β*_2_ represents the change in LVF bias in the deaf group. The intercept was significant (*β*_1_= 17.8 %, *t*_26_= 3.59, *p* = 0.001) indicating the presence of a LVF bias in the hearing group. This LVF bias was not significantly reduced in the deaf group (*β*_2_= -6.42 %, *t*_26_= -0.93, *p* = 0.35). One-sample *t*-test on the LVF values in the group of deaf participants showed a significant LVF bias at the group level (*M* = 11.2%, *t*13 = 2.46, *p* = 0.028).

To test the existence of a LVF bias at the level of the participant we considered the 2 × 2 contingency table formed by the Female or Male responses of the subject to the Female or Male chimeric faces (according to the left part of chimera). Filled data points in **Figure 2** represent the participants for which the χ^2^ statistics for 1 degree of freedom was significant at the *p*-value *α* = 0.05 (bilateral test). The number of participants with LVF bias in the hearing and deaf groups were respectively 6 and 3 (out of 14). One participant in each group showed a RVF bias. To estimate the probability of having 0, 1,…, N significant test under the null hypothesis in a group of *N* = 14 participants we simulated LVF score assuming *n* = 20 draws per participants and a normal distribution of the error with *μ* = 10 and *σ* = 3.6. Note that in theory, with *p* = 0.05, *σ* = √*np*(1-*p*) = 2.24. However because the observed SD in the hearing group was larger we used the observed value which was more conservative. Over 100,000 tests the probability to obtain exactly 3 or 6 significant LVF in a group of 14 individuals was 0.23 and 0.014 respectively. Finding 3 or less significant comparisons occurred in 81 % of the cases. Finding 6 or above occurred only in 1.7% of the cases. To sum-up on the result of this simulation under the null hypothesis of no LVF bias in the population average, finding 3 individuals (out of 14) with a significant LVF bias (as in our group of deaf participants) is likely (*p* = 0.23), in contrast, finding 6 individuals (as in our group of hearing participants) is very unlikely (*p* = 0.017). Although it is an indirect way of testing the presence of a larger bias in the hearing group, these results point in the same direction than the linear model analysis of the LVF bias: that it is stronger on average and more frequent at the individual level in the hearing population.

### Discussion

This experiment was designed to determine if modifications of the hemispheric lateralization for face processing happened in early deaf participants during a gender categorization task with chimeric faces. The results confirmed the presence of a LVF bias in hearing participants that was not significantly reduced in the group of deaf participants. However, the number of participants with LVF bias in the deaf group was not as large as in the hearing group suggesting that the LVF bias might be reduced for some individuals in the population of early deaf adults.

A crucial question when investigating hemispheric dominance by the mean of chimeric faces is the relationship between the LVF bias and the scanning patterns of the participants. Number of studies showed indeed that the left side of the face is investigated first, and for more time, than the right side (Phillips and David, 1997; Butler et al., 2005; Guo et al., 2012). Gazing first at the left of the face would make sense because the left part of the face projects to the right hemisphere when the fixation point is centered on the face. Faster processing of faces in the right hemisphere could then lead to early saccades toward information coming from the left visual hemifield, that is, to the left part of faces.

Some authors argue that the LVF advantage not only reflects the right hemispheric dominance for face processing, but could also arise from the habitual scanning patterns of the participants. Evidence for an effect of the habitual scanning pattern comes from Arabic or Hebrew subjects (right-to-left reading patterns) who show, when compared with English or French readers, a reduced LVF bias (Heath et al., 2005). Moreover when the eye movements are made impossible, the LVF bias is noticeably reduced, although still present (Butler and Harvey, 2006). This LVF advantage could thus arise from the interplay between scanning pattern and hemispheric dominance (see also Butler and Harvey, 2005). This is crucial because it highlights the presence of plasticity arising from different scanning habits during development.

Experiment 2 was designed to confirm the results of experiment 1 but we also recorded eye movements in addition to participant’s responses while deaf and hearing participants performed the gender categorization task with chimeric and normal faces as in Experiment 1. Early deafness has been found to affect the pattern of eye movements in an anti-saccade task (Bottari et al., 2012) and in a task involving judgment of faces’ emotional valence (Watanabe et al., 2011). In addition to the analysis of differences in scanning patterns between deaf and hearing participants in our face categorization task, we also looked for differences in initial fixations and overall exploration of face side and features in an attempt to relate LVF bias to scanning strategy.

## Experiment 2

### Participants

Fourteen deaf participants (six female, mean age = 34.92, *SD* = 8.58), and 14 control hearing subjects (seven female, mean age = 30.84, *SD* = 9.79), contributed to this second experiment. All deaf participants already participated in Experiment 1. Ten out of the 14 control participants of Experiment 1 also took part in this second study.

### Material and Procedure

Sixty faces were presented to the participants: 15 chimeric F/M faces, 15 chimeric M/F faces, 15 average male faces, and 15 average female faces. The stimuli were designed identically to the previous experiment and were presented for 2 s on the screen. Other methodological aspects were identical to Experiment 1. Face image size was 497 × 653 pixels.

Eye movements were recorded from both eyes using a Eyelink 1000 system (SR Research Ltd., Mississauga, ON, Canada) with a 250 Hz sampling frequency. We used a chin-rest to limit head movements. The test phase was preceded by a calibration phase during which participants were instructed to fixate a 0.3° black circle on a gray background which appeared sequentially at five different positions on the screen. During the test phase, a drift correction was made every five trials, in order to realign gaze and screen space, and correct for small head movements. Each trial began by a fixation point. In order to control for starting position effects (Arizpe et al., 2012) the fixation point was placed at the top of the image for half of the trials and at the bottom of the image for the other half.

### Data Analyses

#### Gender Categorization Task

As in Experiment 1, the percentage of correct gender classification for the average female and male faces showed that it was nearly perfect in both groups (deaf: 99.3%; hearing: 99.3%). The mean response time of hearing and deaf participants were almost identical (deaf: *M* = 1082, *SD* = 166 ms; hearing: *M* = 1090 ms, *SD* = 180 ms). The smaller SD in both groups, compared to experiment 1, suggested that inter-individual variability was reduced in this second practice with the gender-recognition task.

**FIGURE 3:**
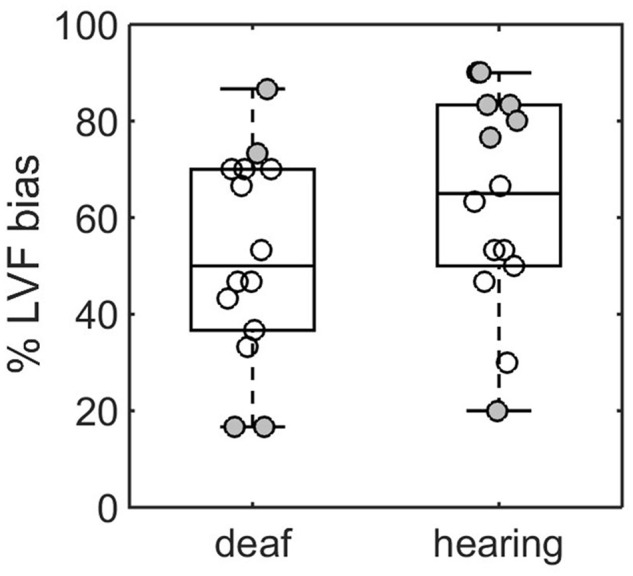
**Visual field bias for deaf and hearing participants in Experiment 2.** Box center and limit give the median, 1st and 3rd quartile. Each data point represents a participant. Filled data point indicates significant bias according to a χ^2^ statistics. Three participants showed a RVF bias (filled dots < 50%).

Although participants seemed more trained to the task, response time was still increased in both groups when judging chimeric faces (deaf: *M* = 1266 ms, *SD* = 304 ms, paired *t*-test, *t*_13_ = -2.99, *p* = 0.01; hearing: *M* = 1354 ms, *SD* = 360 ms, *t*_13_ = -4.32, *p* < 0.001). As in Experiment 1 the average score over the 30 chimeric faces × 100 was used as an index of LVF bias. The boxplot is given in **Figure 3**. Running our GLM on this second dataset we found (*β*_1_= 13.3%, *t*_26_= 2.28, *p* < 0.001) indicating the presence of a LVF bias in the hearing group. This LVF bias was not significantly reduced in the deaf group (*β*_2_= -11.19%, *t*_26_= -1.35, *p* = 0.18). However, the difference from 50% was not significant in the deaf group indicating an absence of LVF bias at the group level (one-sample *t*-test: *M* = 52.14%, *t*_13_ = 0.37, *p* = 0.71).

We tested the LVF bias at the level of the participant as in Experiment 1. Filled data points in **Figure 3** represent the participant for which the χ^2^ statistics for 1 degree of freedom was significant at the *p*-value *α* = 0.05 (bilateral test). The number of participants with LVF bias in the hearing and deaf group were respectively 6 and 2 (out of 14). One participant in the hearing group and two participants in the deaf group showed a RVF bias. Simulated LVF score under null hypothesis assuming *n* = 30 draws per participants and a normal distribution of the error with *μ* = 15 and *σ* = 3.6 indicated that the probability of obtaining exactly 2 or 6 significant LVF score in a group of 14 individuals were 0.28 and 0.003 respectively. Finding three or less significant comparisons occurred in 77% of the cases. Finding six or above occurred only in 0.4% of the cases (*p* = 0.004).

#### Test–Retest Reliability

Because all deaf participants involved in Experiment 2, and 10 of the hearing participants, were also tested in Experiment 1 we estimated the test–retest reliability for the LVF bias. In practice we regressed the LVF score in Experiment 2 using the LVF score in Experiment 1 as a predictor. The coefficient of fidelity *r_xx_* was equal to 0.735 and the regression results gave LVF_2_ = -0.012 + LVF_1_*0.898. The slope of the regression was significant (*t*_22_ = 5.08, *p* < 0.001) while the intercept was not different from 0 (*t*_22_ = -0.1, *p* = 0.92). Thus, the measures from the two gender recognition tasks were clearly related. We then examined the fidelity within both group. For the deaf participants we found *r_xx_*_Deaf_ = 0.77 (LVF_2_ = -0.06 + LVF_1_^∗^0.95; *t*_12_ = 4.9, *p* = 0.0012). For the hearing participants we found *r_xx_*_Hearing_ = 0.56 (LVF_2_ = 0.18 + LVF_1_*0.65; *t*_8_ = 1.91, *p* = 0.09). Reliability of the LVF measure was high in the group of deaf participants and medium in the group of hearing participants. However the smaller number of subjects participating in both experiments in the hearing group make it difficult to draw a firm conclusion on the difference in test–retest reliability between both groups.

Further analysis showed that 5 out of the 6 hearing participants who had a significant visual field bias in Experiment 1, and participated in Experiment 2, showed a similar significant bias in Experiment 2. In the deaf group, 2 out of 4 participants showed a visual field bias in both experiment. Given these results we recomputed the LVF measure of the participants using the results from both experiments when it was possible. These values (see Supplementary Table S1), which presumably best render the participants’ visual bias, were used to examine the relation between scanning strategy and visual field bias in the following section.

#### Eye-Tracking Data

**FIGURE 4:**
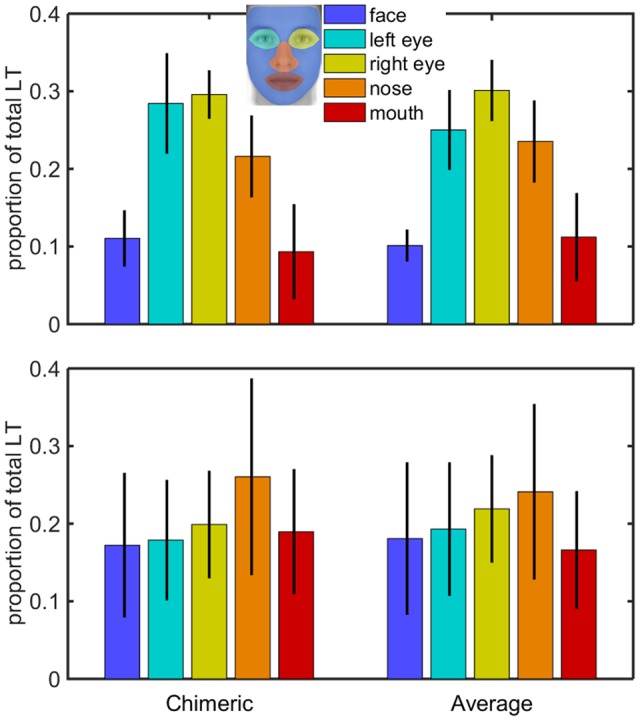
**Proportion of looking time in AOI for the hearing (Top)** and deaf **(Bottom)** group. Black bars give the 95% confidence interval for the mean proportion of looking time.

Raw position signal from the eye-tracker was processed oﬄine. Saccades and fixations were parsed using an algorithm adapted from Engbert and Kliegl (2003). We set the minimum amplitude for saccades to 0.5° of visual angle. Only trials where participants gazed at the face and with the initial fixation located on the fixation point were included in the analyses. The AOI for the mask, used to identify fixation location, were defined *post-hoc* using the full distribution of fixation locations (see Supplementary Figure S1 for details and **Figure 4** for an illustration of the final mask). Five areas of interest were constructed. Supplementary Figure S1 shows the overall proportion of fixations within each AOI. The goal of the analyses was to test whether hearing and deaf participants differed with respect to face scanning and whether individual differences in face scanning could be related to the LVF bias.

##### General oculomotor behavior

In order to check for the existence of differences between hearing and deaf participants with respect to basic aspects of oculomotor behavior, we first analyzed the distribution of fixation duration. The dataset was composed of *N* = 7,751 fixations and was best fitted by a lognormal model with μ = 5.39 (219 ms) and *σ*= 0.51. The mean and standard deviation in the two groups were 5.38 (*n* = 4,158, *SD* = 0.49) and 5.39 (*n* = 3,593, *SD* = 0.54) for the hearing and deaf group respectively. No differences were found between the hearing and deaf groups concerning fixation durations. Saccadic reaction time (i.e., the time between stimulus onset and the onset of a saccade away from the fixation point) was similar in both groups (hearing: *M* = 180 ms, *SD* = 41 ms; deaf: *M* = 200 ms, *SD* = 87 ms, two-sample *t*-test, *t*_26_ = -0.75, *p* = 0.46). The increased SD in the deaf group was due to one subject (D2) who showed very large saccadic RT (*M* = 471 ms, *SD* = 181 ms). No differences were found between the hearing and deaf groups regarding parameters related to saccades (amplitude or velocity). The Supplementary Table S1 shows the extracted parameters for each participant. Knowing that oculomotor behavior was comparable in hearing and deaf participants we analyzed in more detail the scanning pattern of each participant.

##### First saccade

We focused on the first saccade and the subsequent landing position for the first fixation on the stimulus image. To quantify the differences in initial visual attention to the left and right part of the face, we simply divided the number of first fixations landing on the left part of the face by the total number of initial fixations made by participants (i.e., the number of valid trials) and subtracted 0.5 from this ratio. A score of 0.5 thus indicates that all initial fixations were on the left part of the face while a score of -0.5 indicates a right initial fixation bias (IFB). The distribution of our IFB was highly heterogeneous (*M* = 0.16, *SD* = 0.39). As shown in the Supplementary Table S1, most participants had a large positive bias toward the left part of the face (IFB > 0.25, *n* = 14). However, four participants showed a large bias toward the right part of the face (IFB < -0.25). We used a linear model of the form IFB = *β*_1_ + *β*_2_G*_j_* + *𝜀_ij_* where G represented the group and was coded *j* = -0.5 for hearing and *j* = 0.5 for deaf. According to the model the intercept term represents the amount of IFB bias in the whole group and the second term *β*_2_ represents the change in IFB bias due to group. The intercept was significant (*β*_1_= 0.16, *t*_26_= 2.86, *p* = 0.008) confirming the previously observed initial bias toward the left part of faces during face perception tasks. This bias was not influenced by group (*β*_2_= 0.03, *t*_26_= 0.28, *p* = 0.77).

Next, we classified each landing position according to the AOI shown in **Figure 4**. Complementary analyses showed that starting position strongly influenced landing position but this was independent of group and had no influence on the overall IFB (see Supplementary Table S2). Density maps for the location of the initial fixation points depending on starting position are shown in the Supplementary Figure S2.

##### Relative total looking time in AOI

In addition to initial fixations we classified all the fixation durations in our sample according to the predefined AOIs. **Figure 4** shows the mean ratio of looking time to each AOI for the hearing and deaf group as a function of the stimulus type (chimeric, average).

We built a linear model of the ratio of looking time (RLT) of the form RLT = *β*_0_ + *β_j_*AOI*j* + *β_jk_*AOI*_j_* * G*_k_* + *𝜀_ijk_* where AOI was coded *j* = 0, 1, 2, 3, or 4 for the face, left eye, right eye, nose, and mouth areas respectively. Group (G) was coded 1 for hearing participants and 2 for deaf participants. According to the model the intercept term represents the ratio of looking time in the face AOI for the hearing participants. The values of *β_j_* represent the change from this baseline ratio for the left eye, right eye, nose, and mouth AOI for the hearing group. Finally, values of *β_jk_* represent the change from the ratio in the hearing group to the ratio in the deaf group for each AOI. **Table 2** summarizes the results. The ratio of looking time for hearing and deaf participants differed for the face, left eye, right eye, and mouth AOI. Deaf participants spent less time in the eye areas than hearing participants, but they spent more time to the face and mouth area. Framed within the classical ANOVA format we found a main effect of AOI [*F*_(4,270)_ = 9.64, *p* < 0.001] and an AOI x Group interaction [*F*_(4,270)_ = 4.06, *p* < 0.0014]. Complementary analyses showed that stimulus type (average, chimeric) had no effect on the repartition of visual attention toward the faces (see Supplementary Figure S3).

**Table 2:** Result of linear model of the ratio of looking time within AOI.

Group	Mean	*B*	std. Error	*t*-value	*P*
AOI_face_ hearing *(intercept)*	0.105	0.105	0.025	4.17	**<0.001**
AOI_leye_ hearing	0.267	0.162	0.036	4.54	**<0.001**
AOI_reye_ hearing	0.298	0.193	0.036	5.41	**<0.001**
AOI_nose_ hearing	0.226	0.121	0.036	3.39	**0.001**
AOI_mouth_ hearing	0.103	–0.003	0.036	–0.08	0.939
AOI_face_ × deaf	0.177	0.072	0.036	2.01	**0.045**
AOI_leye_ × deaf	0.187	–0.081	0.036	–2.26	**0.024**
AOI_reye_ × deaf	0.208	–0.090	0.036	–2.54	**0.012**
AOI_nose_ × deaf	0.253	0.027	0.036	0.74	0.458
AOI_mouth_ × deaf	0.176	0.073	0.036	2.05	**0.042**

*Residual standard error: 0.1336 on 270 degrees of freedom. Multiple *R*^2^: 0.1791. Adjusted *R*^2^: 0.1517. F-statistic: 6.545 on 9 and 270 DF. *p*-value < 0.001. Bold values indicate significant results.*

Next we calculated an index of visual bias toward the left part of the face similar to the IFB used for the analysis of the first fixation. The distribution of total fixation bias (TFB) was more homogeneous (*M* = -0.004, *SD* = 0.12). As shown in the Supplementary Table S1, most participants had small fixation bias (0.25 < TFB > -0.25). We used the linear model TFB = *β*_1_ + *β*_2_G*_j_* + *𝜀_ij_* where G represented the group and was coded *j* = -0.5 for hearing and *j* = 0.5 for deaf. According to the model the intercept term represents the amount of TFB bias in the whole group and the second term *β*_2_ represents the change in TFB bias due to group. The intercept was non-significant (*β*_1_ = -0.0048, *t*_26_ = -0.195, *p* = 0.85) indicating that the previously observed initial bias toward the left part of faces during face perception tasks is limited to the initial part of exploration. We found no effect of group (*β*_2_ = 0.008, *t*_26_ = -0.16, *p* = 0.87).

#### *Post hoc* Analysis

We analyzed the mean values of LVF index computed using the two experiments (given in Supplementary Table S1) using one sample *t*-test for the hearing and deaf group separately. The LVF bias was significantly different from 0 in the hearing group (*M* = 15.6, *t*_13_ = 2.81, *p* = 0.046) but not in the deaf group (*M* = 6.78, *t*_13_ = 1.38, *p* = 0.189).

Finally, we checked the correlation between the LVF bias and the measures of visual fixation bias (IFB and TFB) and relative time to AOI for the first fixation as well as for the whole set of participants. The correlation of LVF with IFB and TFB were non-significant. The relative time spent to halve faces was not predictive of the response bias in the gender categorization task. Instead, the visual exploration parameters that were most related to the LVF bias were the proportion of fixation time to the left eye (*r* = 0.44, *t*_26_ = 2.54, *p* = 0.017) and the mouth area (*r* = -0.41, *t*_26_ = -2.31, *p* = 0.028). Note that the proportion of fixation time to the left eye area was positively related to LVF while the proportion of initial fixation to the mouth area was negatively related to LVF.

### Discussion

Overall it was more systematic to find a LVF bias at the group level for hearing participants than for deaf participants. If the between-group analysis of behavioral results in Experiments 1 and 2 did not show a global reduction of LVF bias, the number of participants presenting a significant LVF bias was, however, greater in the hearing group than in the deaf group. It suggests some changes in the hemispheric asymmetry, at least in a part of the population of early deaf adults.

The analysis of the fixation patterns revealed interesting findings. First we did not find any significant differences in the left/right repartition of fixations between the two groups, either for the location of the first fixation or for the overall fixation time. However group differences were found in attention to the eye and mouth areas, deaf participants being more attentive to mouth area but less to the eyes than hearing participants. We found a positive correlation between the LVF bias and the relative time spent looking at the left eye, highlighting the importance of this region in the lateral bias. This latter result may explain the reduced LVF bias in deaf participants who spent less time in the eye areas than hearing participants, but spent more time to the face and mouth area. This result makes sense because the left eye is an informative location to decide on the gender of the hemiface. Paying more attention to the mouth area focuses the attention toward the center of the face, thus leading to smaller LVF bias.

## General Discussion

An established fact in perceptual asymmetries is that for many aspects of face identity processing (perception of age, attractiveness, gender or expression) typical individuals attend to information on the right side of the face, leading to a LVF bias (Burt and Perrett, 1997; Butler and Harvey, 2005). This left bias is thought to reflect a right hemisphere advantage for face processing (Yovel et al., 2008). In deaf people, auditory deprivation and use of sign language seem to affect hemispheric lateralization (Bosworth and Dobkins, 1999; Bavelier et al., 2001). To date, very few studies have found modifications of lateralization using face stimuli in deaf people, and only for the processing of facial expressions (McCullough et al., 2005; Letourneau and Mitchell, 2013). The present study is the first to specifically investigate visual field asymmetries for the processing of facial identity using chimeric faces in deaf people. Using a gender categorization task we found that it was less frequent to find a significant LVF in a group of early deaf participants than in hearing controls. This suggests modifications of cerebral lateralization in deaf people for the processing of invariant aspects of faces, suggesting that early deafness, together with the extensive use of signed language, affects not only the processing of facial expressions, but also the core mechanisms underlying face recognition. Our results are in agreement with those obtained by Szelag and Wasilewski (1992), who found using a divided visual field task a reduced LVF bias in congenital deaf children. In their study this absence of visual field advantage seemed to come from a more variable asymmetry in deaf children, with approximately half of deaf children showing a leftward asymmetry and the other half showing a rightward asymmetry. This variability is also present in our experiments where a clear LVF bias was found in fewer deaf participants than in the hearings. This suggests a greater variability in face hemispheric lateralization in deaf people, potentially resulting from an increased role of the left hemisphere relatively to the right.

This variability may be explained by the heterogeneity of the deaf sample; as shown in **Table 1** our deaf participants differ in terms of etiology of deafness, learning age and daily use of sign language, lip reading abilities or the daily use of hearing aid. This heterogeneity may have influenced our results, and can explain a greater variability in terms of brain specialization. In particular the principal language used in the daily life (oral vs. signed) as well as the age of acquisition of sign language could greatly influence the development of visual field asymmetries. It would be of interest to study more directly the impact of sign language in visual field asymmetries for face processing. Another important point to consider is lip-reading ability, as shown by our *post hoc* analysis; there is a negative correlation between fixation time on the mouth area and the amount of left visual bias. It indicates that paying more attention toward the mouth reduces the LVF asymmetries, because it draws attention toward of the center of the face.

The question that arises from our present results is why deafness would affect face processing. One possibility is that during infancy, children have to learn to link auditory and visual inputs to form one unique perceptual object. To identify a person in everyday life, we rely indeed not only on visual processing of faces, but also on the processing of vocal information. In the absence of the auditory modality, visual processing of face should thus become more salient for communication and social interactions. Another possibility is that face processing is influenced by the use of sign language whereby facial expressions convey information about the emotional state of individuals, but also carry linguistic information. Thus, deaf signers have to pay attention to face for both affective and linguistic inputs; it seems thus possible that they develop particular processing mechanisms that allow them to maximize the ability to gather information from faces. Shifts of cerebral lateralization for the processing of facial expressions from right to left have been observed in deaf participants for the processing of facial expressions (Emmorey and McCullough, 2009) and some results show that right hemisphere activation could be reduced for the processing of neutral faces (Weisberg et al., 2012). Interestingly enough, Weisberg et al.’s (2012) results seem to indicate joint effects of auditory deprivation and extensive use of sign language on cerebral activation. To disentangle these two effects, they also tested a group of hearing signers. Activation in the right middle fusiform gyrus for this group was at an intermediate level between deaf signers and hearing non-signers, suggesting a combined effect of the sensory deprivation and use of sign language. However, this study was not specifically designed to test cerebral asymmetry for face processing in deaf, thus additional studies are needed to evaluate more precisely how asymmetry for face processing is modulated by auditory deprivation.

One limitation of our study is that we did not test a group of hearing signers; therefore the question of whether the modifications of visual field asymmetries observed in this study are related to auditory deprivation, expertise with sign language, or a combination of both, remains open. To disentangle the relative influences of sensory deprivation and plasticity resulting from the use of sign language, it will be necessary to investigate asymmetry using chimeric faces in a population of native hearing signers.

One purpose of the present study was to relate the amount of LVF in deaf participants with the left/right scanning behavior of the participants. Previous studies suggest indeed that the LVF bias results not only from a right hemisphere advantage during face processing, but also from the scanning pattern of participants favoring the inspection of the left side of the face. The left side of the face would be investigated first, and longer than the right side (Phillips and David, 1997; Butler et al., 2005; Guo et al., 2012). The LVF bias has been found to be reduced in people who have a reversed scanning pattern such as Hebrew or Arabic readers (Vaid and Singh, 1989; Heath et al., 2005). The reduction of the LVF bias in deaf people could thus come from a reduction of cerebral asymmetry in face areas, from a scanning pattern favoring more the right side of the face, or both. Early deafness has been found to affect the pattern of eye movements (Watanabe et al., 2011; Bottari et al., 2012), even in non-linguistic or non-emotional tasks, suggesting that the habitual gazing pattern toward faces is altered in deaf people. In our study, we found no left/right difference in the scanning patterns of our participants, but there was a difference in the bottom/up repartition of fixations. While deaf participants showed the classical fixation pattern eyes-mouth-nose like hearing controls, the proportion of fixations on the mouth was increased in deaf participants as costs of attention to the eyes. This suggests a tendency in deaf participants to favor more the information contained in the mouth than hearing participants even in non-communication situations. Alterations of gazing behavior in communication situation have been suggested before (Emmorey et al., 2009). In this study the authors found that in a communication situation, beginning ASL signers fixated more the mouth than native deaf ASL signers who fixated preferentially the eyes. Interestingly enough, other results indicate that these alterations of the gazing behavior could extend to the perception of static faces (Letourneau and Mitchell, 2011; Watanabe et al., 2011). Watanabe et al. (2011) used static faces in early deaf and hearing participants and found an increased fixation time on the eyes in the deaf group relatively to the hearing group. This seems at odd with our results, however, this discrepancy can be explain by cultural bias; Watanabe et al.’s (2011) results have been obtained in Japanese participants which makes the comparison difficult to draw as East Asian observers fixate less the eyes than Western Caucasians (Blais et al., 2008; Miellet et al., 2013). In agreement with our results, another recent study (Mitchell et al., 2013) using composite neutral faces showed an increased attention to the bottom of the faces in deaf participants. Taken together, these results suggest that the use of lipreading and attention toward facial expression does affect profoundly the gazing behavior on faces for deaf participants, and extends toward non-communication situations with static and neutral faces.

## Conclusion

This study suggests that early auditory deprivation and/or expertise with sign language affect the processing of faces, by altering hemispheric lateralization and modifying visual attention taken to static faces. These results emphasize the need of more detailed investigations about face perception in early deaf people and the relation between hemispheric lateralization and gazing behavior, as well as the relative influences of auditory deprivation and the use of sign language in this plasticity for face processing.

## Ethics Statement

The experiment was approved by the local ethics committee (“Comité d’éthique des centre d’investigation clinique de l’inter-région Rhône-Alpes-Auvergne”, no. 2014-A00088-39). Subjects signed informed consent before participating in the experiments.

## Author Contributions

Designed the experiments: MD, OP, and DM. Performed the experiments: MD. Analyzed data: MD and DM. Wrote the manuscript: MD, DM, and OP.

## Conflict of Interest Statement

The authors declare that the research was conducted in the absence of any commercial or financial relationships that could be construed as a potential conflict of interest.
