# Supplementary figures and images for: Modifications of Visual Field Asymmetries for Face Categorization in Early Deaf Adults: A Study With Chimeric Faces
Source: Front Psychol. 2017 Jan 20;8:30. doi: 10.3389/fpsyg.2017.00030 (PMC5247456; doi:10.3389/fpsyg.2017.00030)

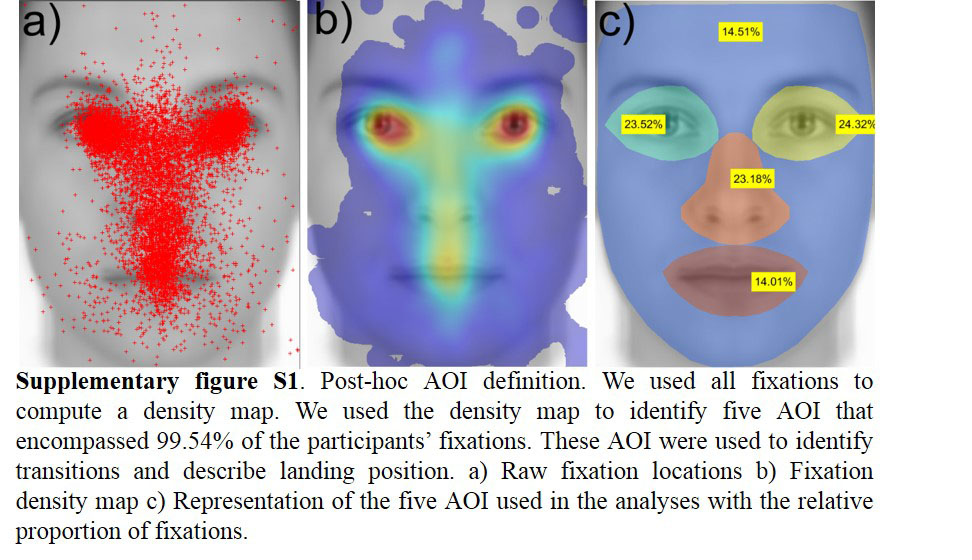

Supplement: Supplementary file 1 [file Image_1.JPEG]

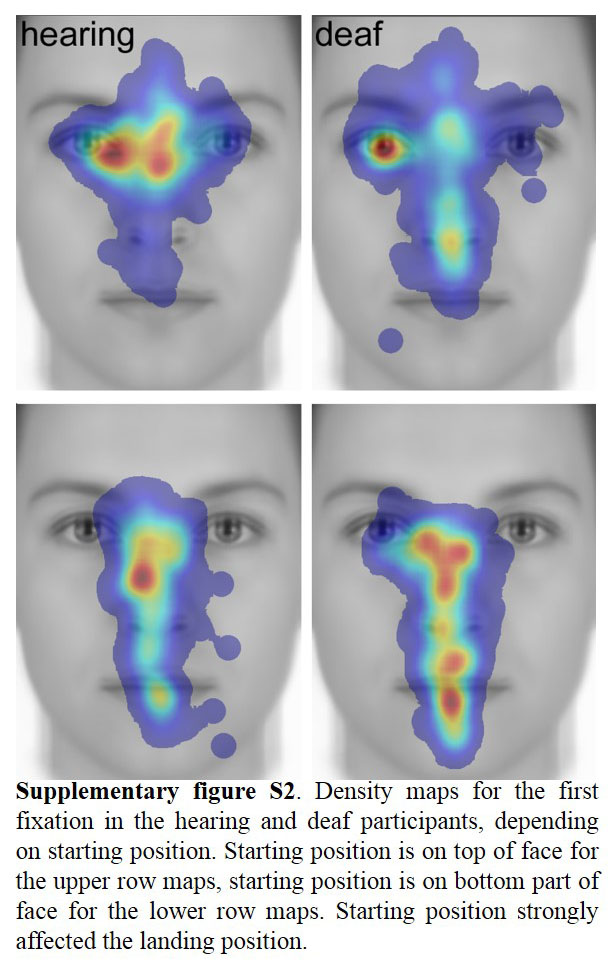

Supplement: Supplementary file 2 [file Image_2.JPEG]

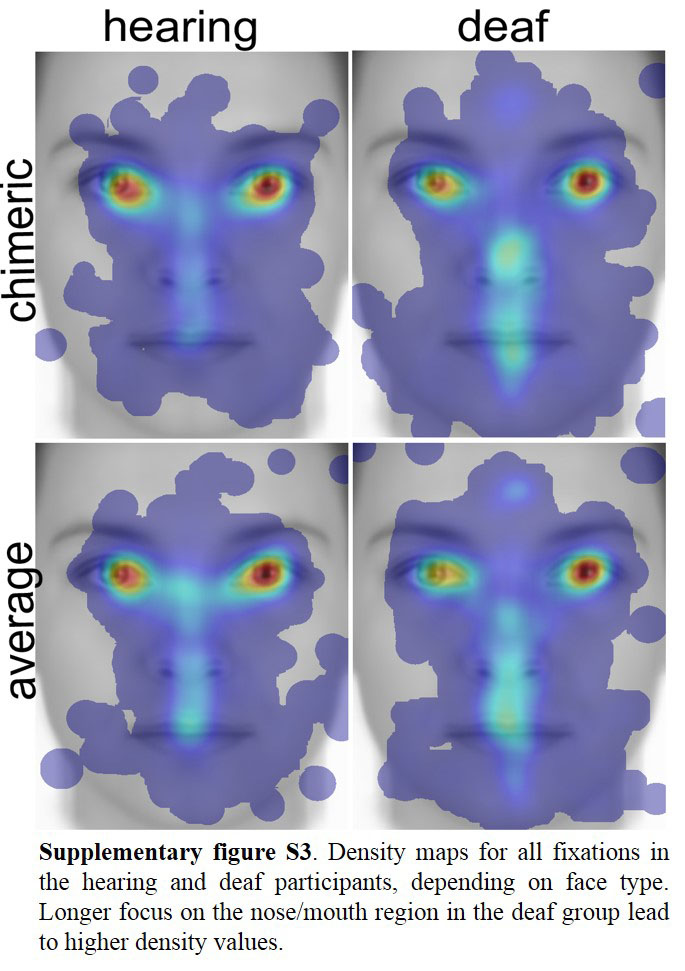

Supplement: Supplementary file 3 [file Image_3.JPEG]
