# Supplementary material for: Modifications of Visual Field Asymmetries for Face Categorization in Early Deaf Adults: A Study With Chimeric Faces
Source: Front Psychol. 2017 Jan 20;8:30. doi: 10.3389/fpsyg.2017.00030 (PMC5247456; doi:10.3389/fpsyg.2017.00030)
Supplement: Supplementary file 4 [file Table_1.docx]

**Supplementary Table S1.** Extracted oculomotor parameters for each participant and each group.

|  | Gender recogition task | | | First saccade | | | All fixations | | | |  |
| --- | --- | --- | --- | --- | --- | --- | --- | --- | --- | --- | --- |
| Participant | N chimeric trial | LVF* | Resp Time | NumFirst | RT | SD RT | IFB | Num Fix | Fix Dur | TFB | |
| H1 | 50 | 22 | 1327 | 51 | 190 | 34 | 0.42 | 372 | 174 | 0.07 | |
| H2 | 50 | 35 | 1033 | 57 | 212 | 56 | 0.43 | 208 | 282 | 0.09 | |
| H3 | 30 | 40 | 937 | 54 | 141 | 47 | 0.45 | 331 | 223 | 0.06 | |
| H4 | 30 | -3 | 1471 | 48 | 142 | 32 | -0.2 | 334 | 208 | -0.11 | |
| H5 | 50 | 32 | 958 | 55 | 148 | 42 | 0.47 | 352 | 193 | 0.12 | |
| H6 | 50 | 33 | 811 | 54 | 289 | 115 | 0.22 | 234 | 244 | 0.06 | |
| H7 | 50 | 7 | 1667 | 42 | 199 | 55 | 0.47 | 265 | 239 | 0.08 | |
| H8 | 30 | -30 | 1707 | 50 | 167 | 28 | -0.23 | 377 | 166 | -0.02 | |
| H9 | 50 | 6 | 1207 | 53 | 162 | 63 | 0.03 | 241 | 264 | 0.02 | |
| H10 | 30 | 3 | 1690 | 54 | 225 | 74 | -0.33 | 319 | 233 | -0.24 | |
| H11 | 50 | 0 | 1476 | 57 | 170 | 71 | 0.17 | 315 | 223 | -0.01 | |
| H12 | 50 | 37 | 771 | 46 | 142 | 42 | -0.27 | 256 | 187 | -0.07 | |
| H13 | 50 | 3 | 1867 | 50 | 167 | 40 | 0.02 | 312 | 237 | -0.13 | |
| H14 | 50 | 35 | 1657 | 59 | 181 | 43 | 0.38 | 233 | 306 | 0.07 | |
| D1 | 50 | -1 | 1648 | 45 | 192 | 57 | 0.32 | 296 | 251 | 0.03 | |
| D2 | 50 | 20 | 1501 | 28 | 471 | 182 | 0.28 | 86 | 221 | 0.05 | |
| D3 | 50 | -22 | 1511 | 58 | 189 | 84 | 0.42 | 135 | 262 | 0.06 | |
| D4 | 50 | 1 | 1579 | 44 | 235 | 47 | 0.48 | 171 | 249 | 0.19 | |
| D5 | 50 | 21 | 1524 | 47 | 280 | 119 | -0.13 | 286 | 226 | -0.03 | |
| D6 | 50 | 33 | 1412 | 52 | 147 | 41 | 0.45 | 291 | 230 | 0.04 | |
| D7 | 50 | -3 | 1231 | 56 | 148 | 60 | 0.32 | 257 | 242 | 0.14 | |
| D8 | 50 | 1 | 1352 | 43 | 159 | 55 | 0 | 243 | 279 | -0.27 | |
| D9 | 50 | 1 | 2015 | 42 | 144 | 39 | 0.37 | 410 | 179 | 0.07 | |
| D10 | 50 | 14 | 1660 | 57 | 153 | 51 | -0.37 | 254 | 197 | -0.10 | |
| D11 | 50 | 9 | 1516 | 50 | 150 | 36 | 0.17 | 318 | 200 | 0.09 | |
| D12 | 50 | -29 | 1552 | 52 | 134 | 41 | 0.12 | 362 | 192 | -0.11 | |
| D13 | 50 | 36 | 1560 | 58 | 202 | 46 | 0.4 | 266 | 216 | 0.08 | |
| D14 | 50 | 15 | 1238 | 30 | 184 | 42 | -0.35 | 237 | 239 | -0.35 | |

*values for the LVF bias are the total bias measured over the two experiments when it was possible
