# Supplementary material for: Modifications of Visual Field Asymmetries for Face Categorization in Early Deaf Adults: A Study With Chimeric Faces
Source: Front Psychol. 2017 Jan 20;8:30. doi: 10.3389/fpsyg.2017.00030 (PMC5247456; doi:10.3389/fpsyg.2017.00030)
Supplement: Supplementary file 5 [file Table_2.DOCX]

**Supplementary Table 2.** Frequency of first saccades toward each AOI as function of starting position and in both groups of participants.

| Group | Position | Face | Left eye | Right eye | Nose | Mouth |
| --- | --- | --- | --- | --- | --- | --- |
| Hearing | top | 0.3233 | 0.3289 | 0.1178 | 0.2195 | 0.0106 |
|  | bottom | 0.1534 | 0.0925 | 0.0425 | 0.5432 | 0.1684 |
| Deaf | top | 0.2847 | 0.2452 | 0.0516 | 0.3762 | 0.0423 |
|  | bottom | 0.1200 | 0.1560 | 0.0046 | 0.4411 | 0.2782 |
